# Dynamic Change of Carbon and Nitrogen Sources in Colonized Apples by *Penicillium expansum*

**DOI:** 10.3390/foods11213367

**Published:** 2022-10-26

**Authors:** Di Gong, Yang Bi, Yuanyuan Zong, Yongcai Li, Edward Sionov, Dov Prusky

**Affiliations:** 1 College of Food Science and Engineering, Gansu Agricultural University, Lanzhou 730070, China; 2 Department of Food Science, Agricultural Research Organization, The Volcani Center, Rishon LeZion 7505101, Israel; 3 Department of Postharvest Science, Agricultural Research Organization, The Volcani Center, Rishon LeZion 7505101, Israel

**Keywords:** *Penicillium expansum*, apple, fungal infection, carbon source, nitrogen source

## Abstract

*Penicillium expansum* is a necrotrophic pathogen, which actively kills host cells and obtains nutrients from dead cells to achieve infection. However, few reports have elucidated the differential levels of carbon and nitrogen sources over increasing distances of the leading edge in fungal colonized fruit tissues during colonization. Our results showed that the highest consumption of sucrose and fructose, as well as the accumulation of glucose, were found in the decayed region of *P. expansum*-colonized ‘Delicious’ apple fruit compared with the healthy region at the leading edge and the healthy region 6 mm away from the leading edge. As nitrogen sources, the contents of methionine, glutamate, leucine, valine, isoleucine and serine were the lowest in the decayed region compared with the healthy regions during colonization. In addition, the titratable acidity, oxalic acid, citric acid, succinic acid and malic acid showed the highest accumulation in the decayed region compared with the healthy regions. *P. expansum* colonization induced the accumulation of saturated fatty acids in the decayed region, while the level of unsaturated fatty acids was the lowest. These changes were not observed in the healthy regions. These results indicated that *P. expansum* kills cells in advance of its colonization in order to obtain the nutrients of the apple tissue from the distal leading tissue of the colonized apple. It is understood that more carbon and nitrogen sources are required for fungal colonization, and a stronger defense response against colonization occurred in the fruit, causing the transit of nutrients from the distal tissue to the infected sites.

## 1. Introduction

*Penicillium expansum* is one of the major postharvest pathogens, which causes the blue mold in pomes, stones and berries [1]. As a necrotrophic fungal, *P. expansum* kills the host cells in the colonized sites by secreting extracellular enzymes and acquires nutrients from these dead cells [2]. Subsequently, due to more demands for nutrients to facilitate colonization, the healthy tissues around the colonized sites are gradually destroyed, causing the expansion of the decay [3,4,5]. Therefore, the development of decay in fruit is a dynamic process that gradually develops outward from the initial colonized sites to the healthy tissue around it during fungal colonization [6,7].

During the process of colonization, fungi usually face nutrient limitations. The sugars and amino acids in fruit are considered as major carbon and nitrogen sources for fungi, which are utilized by sugar or amino acid transporters located in the fungal hyphae to achieve the fungal colonization of fruits [8]. The contents of sucrose, fructose, methionine and glutamate were decreased in the decayed tissue of *P. expansum*-infected apple fruit [9]. A reduced sucrose content and increased glucose and fructose content, as well as the total soluble solid content, were observed in the healthy tissue of *Monilinia fructicola*-infected peach fruit [10]. Moreover, a decreased glucose and sucrose content was found in the healthy tissues of citrus fruit after *P. digitatum* infection [11]. In addition, higher contents of amino acids were found in the healthy tissue of *P. expansum*-infected apple fruit [12].

Organic acids contribute to the pH of fruit [13]. Compared with the healthy tissue of fruit infected by *P. digitatum* and *P. expansum*, higher concentrations of malic and citric acid were found in the tissue at the leading edge of apple and orange fruit [14]. Moreover, higher concentrations of citric acid accumulated in the decayed region of *P. expansum*-infected apple fruit and *P. digitatum*-infected grapefruit [13]. Our previous results indicated that *P. expansum* inoculation decreased the titratable acid content in the healthy tissue of apple fruit [15]. Fatty acids form the main composition of the cell membrane in fruit tissue and, together with the high level of unsaturated fatty acids (USFAs)/saturated fatty acids (SFAs), contribute to maintaining the fluidity and integrity of the cell membrane [16,17]. However, fungal infection reduced the ratio of USFAs/SFAs in the healthy tissue of fruit, resulting in an impaired integrity of the cell membrane of the host [18,19], which enabled the release of primary metabolites from the cells and their use for fungal colonization. *Phomopsis longanae* infection decreased the integrity of the cell membrane by increasing the levels of palmitic acid and stearic acid and reducing the levels of oleic acid, linoleic acid and linolenic acid in the healthy tissue of longan fruit [20,21]. In addition, lower contents of oleic acid and linoleic acid were observed in the decayed tissue of *P. expansum*-infected apple fruit [9].

The latest results illustrated differences in the contents of sugars, organic acids, amino acids and fatty acids in the decayed tissue at the leading edge of the center region of decay in *P. expansum*-infected ‘Fuji’ apple fruit [9]. However, the changes in these essential compounds between the colonized and healthy regions of fruit during fungal colonization are poorly understood. Therefore, the objective of the present study was to analyze changes in the contents of major sugars, organic acids, amino acids and fatty acids in three different regions of the ‘Delicious’ apple fruit during *P. expansum* inoculation, including the decayed tissue at the leading edge of decay, the healthy tissue at the leading edge and the healthy tissue 6 cm away from the leading edge, in order to understand the contributions of these essential metabolites in fruit to fungal colonization. Understanding the dynamic change in carbon and nitrogen sources in *P. expansum*-colonized apples can help us to further investigate the infection mechanism of *P. expansum*, contributing to the control of blue mold in apple fruit.

## 2. Materials and Methods

### 2.1. Fruit

Apple fruit (*Malus domestica* Borkh. cv. Delicious) was harvested from a commercial orchard in Jingtai, Gansu Province, China. Fruits of a uniform size and similar maturity that were free from wounds and fungal infection were collected from different apple trees. Subsequently, the fruits were individually covered with foam net bags, put into corrugated boxes and transferred to the lab on the day of harvest and stored in a cool room (5 ± 2 °C, with a RH 80–90%) until further use.

### 2.2. Preparation of the Spore Suspension

*P. expansum* (T01) was supplied by Prof. Shiping Tian of the Institute of Botany, Chinese Academy of Sciences. The strain was grown on potato dextrose agar plates (PDA) at 25 °C for 7 days. For the spore collection, the plates were flooded with 5 mL sterile water, and the spores removed by gentle rubbing with a sterile glass rod, followed by filtration through four layers of sterile cheesecloth. The concentration of the spore suspension was determined using a hemocytometer and then diluted to a final concentration of 1 × 10^6^ spore mL^−1^.

### 2.3. Fruit Inoculation

The fruits were taken out of cool storage. After warming to an ambient temperature (22 ± 2 °C) for 24 h, they were washed with tap water and surface-sterilized in 1% NaClO_3_ for 2 min, and then washed again with sterile water and dried at an ambient temperature for 1 h. Two wounds were created with a sterile nail (2 mm depth and 2 mm diameter) on opposite sides of the apple along the equator on each fruit before inoculation. Then, the apple fruit was inoculated with 10 μL of spore suspension in each wound. Sterile water inoculation was used as a control. After air-drying, all the fruits were placed into polyethylene bags and stored at an ambient temperature (22 ± 2 °C, RH 55–65%).

### 2.4. Sampling

**Figure 1:**
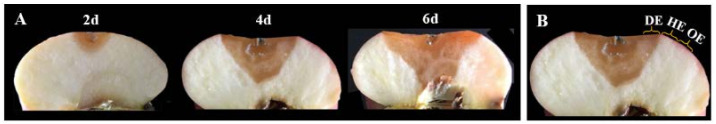
Pictures of lesion development in *P. expansum*-colonized apple fruit at 2, 4 and 6 days of colonization (**A**). Three regions of the tissues were collected (**B**). DE, the decayed region at the leading edge; HE, the healthy region at the leading edge; OE, the healthy region 6 mm away from the leading edge.

According to the method of Gong et al. [15], at 2, 4 and 6 days after inoculation, 3 mm-thick tissues of the peel and pulp in the decayed region at the leading edge (DE), the healthy region at the leading edge (HE) and the healthy region 6 mm away from the leading edge (OE) were sampled from each fruit, respectively, as shown in Figure 1. In the experiment, tissues from the same region were blended, and 3 g of them were weighted and packaged with tinfoil and then immediately frozen in liquid nitrogen. The samples were stored at −80 °C until the time of analysis. A total of 36 apple fruits were contained in each group, and 12 apple fruits in each group were used for each sampling time. For each measurement, 3 packages of the samples of the same tissues were used, and 3 replicates were performed for the measurements.

### 2.5. Measurement of the Total Soluble Solids (TSS) and Titratable Acidity (TA) Contents

The TSS content of the fresh tissues was determined using a digital refractometer (TD-45, Hangzhou, China) and expressed as %. Ten grams of frozen tissues were ground and diluted to 100 mL with distilled water for the TA determination. Ten mL of solution was taken and titrated with 0.2 M NaOH until a pH of 8.2 was reached, and the result was expressed as % [22].

### 2.6. Analysis of the Sugar and Organic Acid Contents

The sugars and organic acids were extracted using the method of Zhang et al. [23] with modifications. Three grams of frozen tissue were finely ground and homogenized in the presence of 10 mL of cold HPLC-grade ethanol (Sigma-Aldrich Products, St. Loui, MO, USA) and then incubated at 35 °C for 20 min and centrifuged at 10,000× *g* for 10 min. The supernatant was transferred to a tube, and the residues were extracted again using the extraction protocol. Supernatants were added up to volume of 25 mL with ethanol. Subsequently, 1 mL of the solution was dried using nitrogen at 55 °C, and then the residue was resuspended in 0.5 mL of distilled water and filtered through a 0.22 μm syringe filter (Agela Technologies, Torrance, CA, USA). The filtered solution was used for the sugar and organic acid analysis by ACQUITY HPLC (Waters Beckman Coulter Inc., Milford, MA, USA).

The sugars (fructose, glucose and sucrose) were analyzed as described by Gancedo and Luh [24], with modifications. A chromatographic separation of the sugars was carried out using a ZORBAX carbohydrate column (4.5 μm, 4.6 mm × 250 mm, Agilent GL Sciences Inc., Santa Clara, CA, USA). The mobile phase consisted of acetonitrile:water (80:20, *v*/*v*), and the flow rate was 1.4 mL min^−1^. Eluted peaks were detected with a refractive index detector, and the data were analyzed with a Waters Empower system. Organic acids (oxalic acid, citric acid, succinic acid and malic acid) were analyzed as described by López-Hernández et al. [25], with modifications. The chromatographic separation of the organic acids was carried out using an ODS C18 column (4.6 mm × 250 mm, Waters Beckman Coulter Inc., Milford, MA, USA). The mobile phase consisted of (NH_4_)_2_HPO_4_ and the flow rate was 0.5 mL min^−1^. Eluted peaks were detected with a refractive index detector, and the data were analyzed with a Waters Empower system. The sugars and organic acids were detected at 210 nm. The peak area of each sugar and organic acid was quantified by comparison with the calibration curve of the corresponding standards. The concentrations of sugars and organic acids were expressed as mg g^−1^.

### 2.7. Analysis of the Amino Acid Contents

The amino acid contents were analyzed using the method of Li et al. [26], with some modifications. One gram of frozen tissue was ground and homogenized with 10 mL of 5% (*v*/*v*) sulfosalicylic acid. Subsequently, the mixture was centrifuged at 12,000× *g* for 20 min at 4 °C, and the supernatant was transferred to a new tube. The amino acid content was quantified by reference to 17 amino acid mixed standards using a Hitachi L-8800 Amino Acid Automatic Analyzer (Hitachi-Hitec, Omuta-shi, Japan) equipped with a Hitachi custom ion exchange resin (4.6 mm ID × 60 mm). The quantity of each amino acid was expressed as mg kg^−1^. 

### 2.8. Analysis of the Fatty Acid (FA) Contents

The FAs were analyzed using the method of Valero-Garrido et al. [27], with some modifications. Three grams of frozen tissue were ground, and 9 mL hexane:isopropanol (3:2, *v*/*v*) and 100 μL heptadecanoic acid were added into it as an internal standard. The mixture was heated at 85 °C for 1 h and then cooled and supplemented with 1 mL hexane. After 1 h, 100 μL of supernatant was adjusted to 1 mL of hexane. The lipids were quantified as the FA methyl esters using a Trace 1310 gas chromatograph (ThermoFisher Scientific Inc., Waltham, MA, USA) equipped with a TG-5MS column (30 m × 0.25 mm × 0.25 μm, ThermoFisher Scientific Inc., Waltham, MA, USA). The initial oven temperature was 85 °C, which was held for 2 min and then increased by 25 °C min^−1^ to 220 °C, by 5 °C min^−1^ to 250 °C and by 2 °C min^−1^ to 270 °C, and then held for 5 min. The injector and detector were maintained at 290 °C. The splitter ratio was 50:1, the injection volume 1 μL, and the hydrogen flow was 1.2 mL min^−1^. The FA content was quantified against the internal standard, and the quantity of each FA was expressed as mg kg^−1^. The double bond index (DBI) was calculated using the following equation: DBI = ([18:1] + 2 × [18:2] + 3 × [18:3])/([16:0] + [18:0]).

### 2.9. Statistical Analysis

All the data are expressed as the mean ± standard error of the 3 replicates. A statistical analysis was performed using SPSSv19.0 (SPSS Inc., Chicago, IL, USA). Significant differences between the samples were calculated using one-way ANOVA followed by Duncan’s test at *p* < 0.05.

## 3. Results and Discussion

### 3.1. Effects of P. expansum Colonization on the Contents of TSS and Individual Sugars in Different Regions of the Apple Fruit

**Figure 2:**
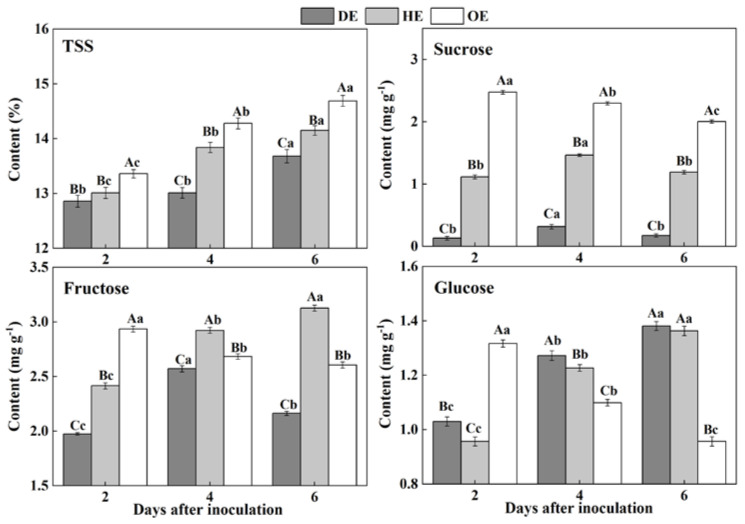
The contents of TSS, sucrose, fructose and glucose in different regions of *P. expansum*-inoculated apples during incubation. DE: the decayed tissue at the leading edge; HE: the healthy tissue at the leading edge; OE: the healthy tissue 6 mm away from the leading edge. The error bar indicates the standard error (±SE). Capitals indicate a significant difference between different groups at the same time point, and small letters indicate a significant difference in the same group at the different time points (*p* < 0.05).

Sugars are considered as an important form of carbohydrates in fruit, which are stored in the vacuoles of cells in harvested fruit [28]. During colonization, sugars are taken up from the fruit by pathogens as carbon sources to participate fungal development [29]. In the present study, the contents of TSS increased in the three regions of fruit, but the TSS content declined from the distal healthy region to the decayed region of the fruit during incubation. The sucrose content in the DE region was 88.1% and 94.6% lower than that in the HE and OE regions by the 2nd day of inoculation, respectively (Figure 2). The accumulation of fructose declined in the DE region, being 30.8%, which was 17% lower than that in the HE and OE regions by the 6th day of inoculation, respectively (Figure 2). A different pattern was observed in the consumption of glucose. The content of this sugar was lower in both the DE and HE regions compared to the OE region on the 2nd of inoculation. However, after that, the glucose content increased in both the DE and HE regions and declined in the OE region (Figure 2). These results may indicate that *P. expansum* mostly consumed sucrose and fructose as carbon sources for colonization.

The accumulation of TSS, sucrose, fructose and glucose in the decayed region is closely related to the disruption of the fruit’s cell structure caused by fungal colonization. Various extracellular enzymes are secreted into the decayed tissue by the fungus to disassemble the cell wall of the fruit during colonization [30], causing the degradation of the cell wall polysaccharide and the increase in the soluble sugar contents [31]. Moreover, fungal colonization facilitates the breakdown of the vacuole of fruit [32], which causes the leakage of the stored fructose and glucose into to the apoplasmic space in the fruit cells [33]. Our results showed that fungus prefers to degrade or directly use sucrose as a carbon source during colonization, which caused a rapid decrease in the sucrose content in the decayed tissue. The cell wall degradation enzymes secreted by the tips of the fungal mycelium degrade the cellular components of fruit during colonization, leading to the accumulation of TSS, sucrose, fructose and glucose in the healthy regions of the leading edge. Moreover, fungal infection improves the ethylene production of fruit, which accelerates the polysaccharide degradation of the fruit [34], elevating the sugar contents. Additionally, the higher accumulation of glucose and fructose in the healthy tissues of the leading edge may be the results of the transportation of these sugars from the distal tissues in order to defend against pathogen infection [35,36]. Sucrose can be degraded to create glucose and fructose [37], which also enhanced the contents of fructose and glucose in the healthy tissues of the leading edge. The increase in the TSS in the healthy tissues away from the leading edge may be due mainly to the polysaccharide degradation caused by fungal-induced ethylene production. Moreover, the decrease in the sucrose, fructose and glucose contents may relate to the increase in the respiration of the inoculated fruit, which leads to more demands for energy and a reduced activity for the defense response of the fruit.

### 3.2. Effects of P. expansum Colonization on the Contents of Amino Acids in Different Regions of Apple Fruit

**Figure 3:**
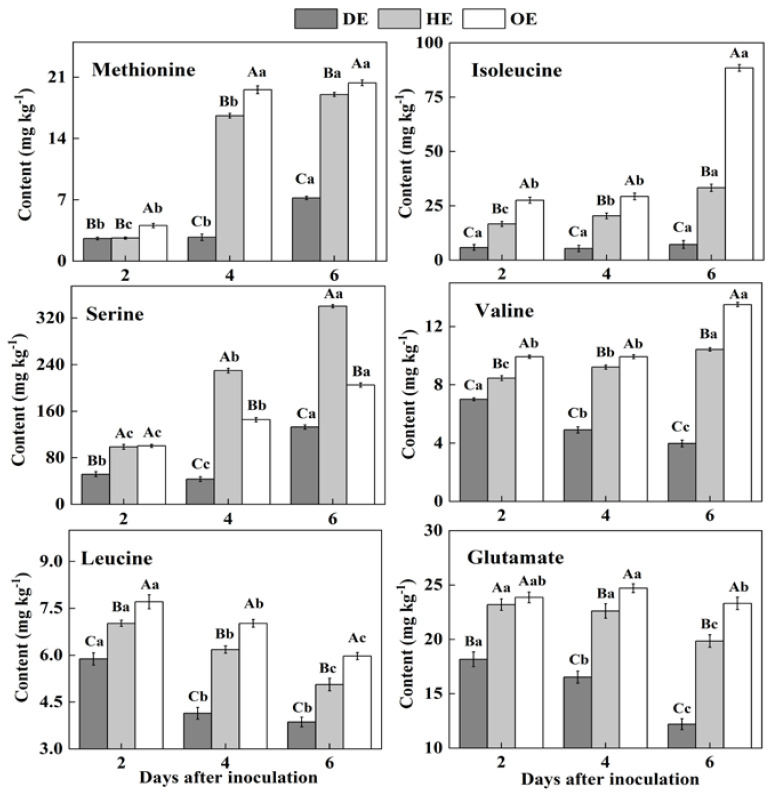
The decreased contents of amino acids in the decayed region compared with the healthy regions of *P. expansum*-inoculated apples during incubation. DE: the decayed tissue at the leading edge; HE: the healthy tissue at the leading edge; OE: the healthy tissue 6 mm away from the leading edge. The error bar indicates the standard error (±SE). Capitals indicate a significant difference between different groups at the same time point, and small letters indicate a significant difference in the same group at the different time points (*p* < 0.05).

**Figure 4:**
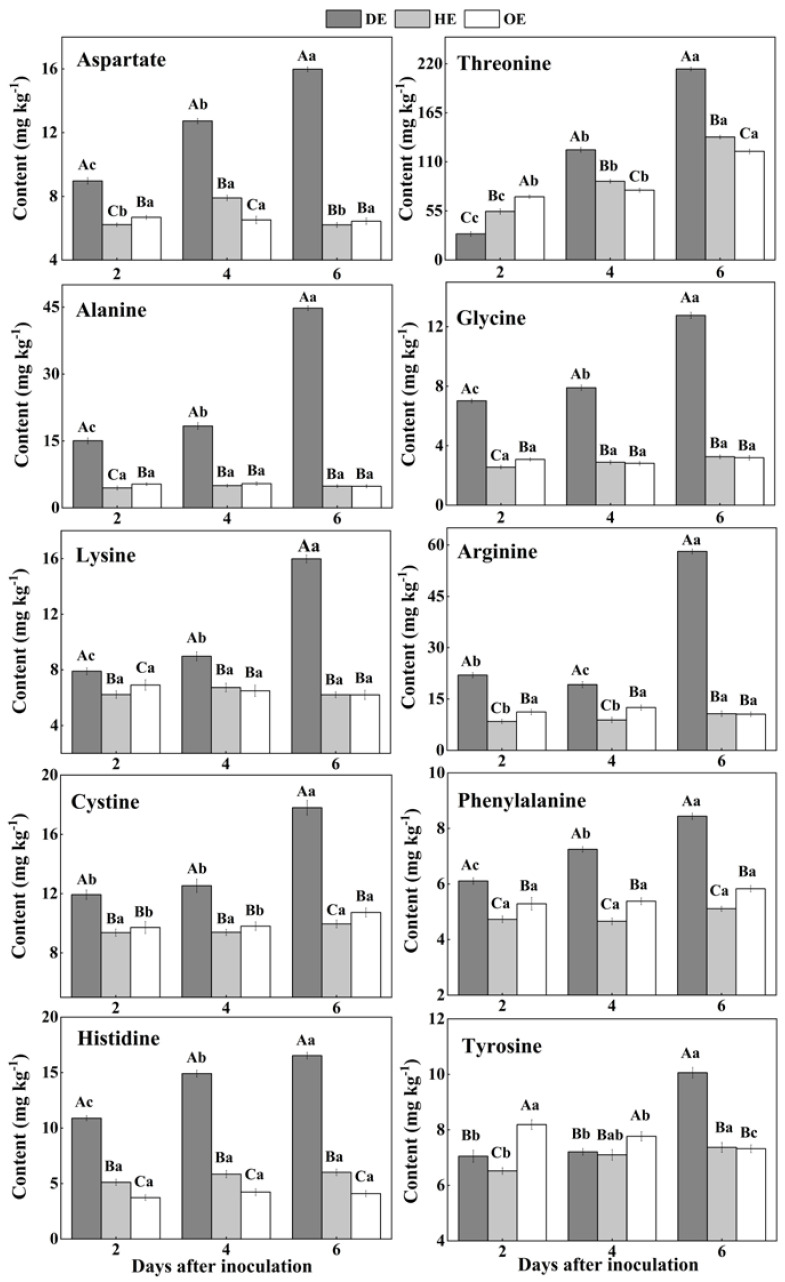
The increased contents of amino acids in the decayed region compared with the healthy regions of *P. expansum*-inoculated apples during incubation. DE: the decayed tissue at the leading edge; HE: the healthy tissue at the leading edge; OE: the healthy tissue 6 mm away from the leading edge. The error bar indicates the standard error (±SE). Capitals indicate a significant difference between different groups at the same time point, and small letters indicate a significant difference in the same group at the different time points (*p* < 0.05).

In fruit, amino acids are mainly stored in the vacuole, and they take part in various biological and metabolic processes in the fruit. Moreover, pathogenic fungi utilize amino acids as nitrogen sources during colonization [38,39]. Two differential accumulations of amino acids were observed in the colonized tissue. While there was a decreased accumulation of methionine, isoleucine, serine, valine, leucine and glutamate, another group of amino acids, including aspartate, threonine, lysine, arginine, alanine, glycine, cystine, phenylalanine, histidine and tyrosine, showed an increased accumulation in comparison to the healthy tissue (Figure 3 and Figure 4). Except for leucine, methionine, glutamate, valine, isoleucine and serine contents increased in the healthy tissues (HE and OE) during incubation (Figure 3). The range of the decrease comparing the healthy with the decayed tissues in the contents of these amino acids was from 0- to 11.3-fold. The observations of the lowest contents of methionine, glutamate, leucine, valine, isoleucine and serine in the colonized tissue, in comparison to the healthy tissues, indicate the consumption of these amino acids by the pathogen (Figure 3).

On the contrary, after the 2nd day of inoculation, the highest content of aspartate was observed in the decayed region, which increased by 55.4% and 75.9% compared with the HE and OE regions by the 6th day of inoculation, respectively (Figure 4). The threonine, lysine, arginine, alanine, glycine, cystine and tyrosine contents showed no significant difference between the HE and OE regions during colonization. By the 6th day of inoculation, the range of the contents of threonine, lysine, arginine, alanine, glycine, cystine and tyrosine comparing the healthy with the decayed tissues was from 0- to 8.3-fold (Figure 4). The increased accumulation of amino acids suggested a metabolic response of the fruit to the pathogen, inducing a specific accumulation in the apple tissues.

Pathogenic fungi secrete proteases into the host during colonization, which causes proteolysis at the infected sites of the fruit, leading to the accumulation of amino acids in the decayed tissues of fruit [40]. Moreover, due to the fact that most of the amino acids are stored in the vacuole of the fruit [39], we hypothesized that fungal colonization may destroy the vacuole of the fruit, causing the release of amino acids into apoplasmic space and their accumulation in the decayed region. In the present study, the lowest contents of methionine, isoleucine, serine, valine, leucine and glutamate and the accumulated contents of aspartate, threonine, lysine, arginine, alanine, glycine, cysteine, phenylalanine and histidine were observed in the decayed tissue (Figure 3 and Figure 4). It has been indicated that fungi can selectively take up amino acids from fruit as nitrogen sources in order to meet the demands for colonization [41]. Therefore, the decrease in those amino acids in the decayed region may be more beneficial for *P. expansum* colonization. Additionally, the accumulation of methionine, isoleucine, serine, valine, glutamate and aspartate in the healthy tissue of the leading edge may be involved in their participation in the defense response of the fruit [42]. Methionine is the precursor of ethylene biosynthesis in fruit, and ethylene, as a signal, participates in the defense resistance of fruit [43]. Moreover, serine, isoleucine, aspartate and valine, as signals, can also contribute to the resistance response of fruit [44,45,46]. In addition to being related to the defense response, the accumulations of methionine, isoleucine, serine, valine, glutamate and aspartate during colonization may be also related to infection-induced ethylene production, which causes protein degradation. 

### 3.3. Effects of P. expansum Colonization on the Contents of TA and Individual Organic Acids in Different Regions of the Apple Fruit

**Figure 5:**
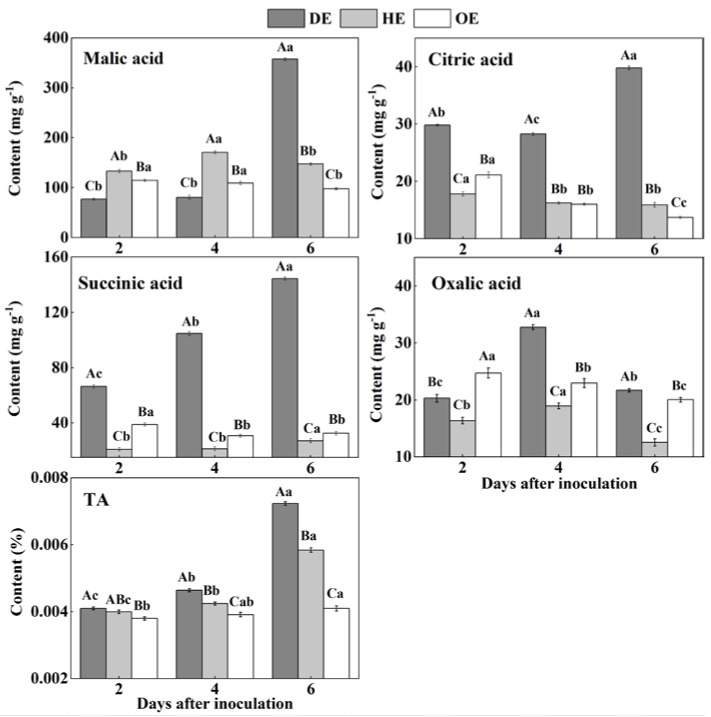
The contents of TA, malic acid, citric acid, succinic acid and oxalic acid in different regions of *P. expansum*-inoculated apples during incubation. DE: the decayed tissue at the leading edge; HE: the healthy tissue at the leading edge; OE: the healthy tissue 6 mm away from the leading edge. The error bar indicates the standard error (±SE). Capitals indicate a significant difference between different groups at the same time point, and small letters indicate a significant difference in the same group at the different time points (*p* < 0.05).

Organic acids such as citric acid, malic acid and succinic acid participate in the TCA cycle and are mostly stored in the vacuole of fruit [47]. The TA content accumulated mostly in the decayed tissue, reaching the most significant difference by the 6th day of inoculation, which was 23.80% and 76.77% higher than that in the HE and OE regions, respectively (Figure 5). The HE region had the highest content of malic acid in the first 4 days of inoculation compared with the other two regions, but it was the highest in the DE region by the 6th day of inoculation, increasing by 1.4- and 2.7-fold compared to that in the HE and OE regions, respectively (Figure 5). By the 6th day of inoculation, the citric acid content in the DE region was 1.5- and 1.9-fold higher than that in the HE and OE regions, respectively (Figure 5). The succinic acid content increased by 4.3- and 3.4-fold in the DE region compared to that in the HE and OE regions, respectively (Figure 5). The content of oxalic acid showed a higher amount in the decayed tissues, which increased by 73.1% and 42.7% compared to that in the HE and OE regions on the 4th of inoculation, respectively (Figure 5). These results indicated that *P. expansum* increased the accumulation of organic acid in the decayed tissues compared with the healthy tissues of the infected fruit.

The accumulations of TA, malic acid, citric acid, succinic acid and oxalic acid in the decayed region of the fruit are related to the breakdown of the fruit vacuole by the fungal infection. Due to the fact that most of the organic acids are stored in the vacuole, fungal colonization destroys the membrane of the vacuole, leading to the leakage of these organic acids into the apoplasmic space and causing the accumulation of these organic acids in the decayed tissues [47]. Malic acid predominates among the organic acids of apple fruit, which can enter into the gluconeogenesis pathway to produce glucose [48]. This may explain the accumulation of glucose and the reduction in malic acid in the decayed region. *P. expansum* is described as an “acidifying fungi” that reduces the host pH by secreting gluconic and citric acid during colonization, which may contribute to citric acid accumulation in the decayed tissues [49]. Moreover, the citric and succinic acids in fruit are mainly produced by the TCA cycle [48]. Fungal colonization may improve the TCA cycle in the decayed region of the fruit, increasing the contents of citric and succinic acids. The oxalic acid in fruit is derived from the TCA cycle and ascorbic acid degradation [50]. The accumulation of oxalic acid in the decayed tissues may relate to the increased TCA cycle and induction of the antioxidant system of ascorbic acid by the pathogen infection. In addition, the accumulation of TA and organic acids in the healthy tissues of the leading edge is the result of the enhanced TCA cycle and tissue acidification caused by fungal colonization. Furthermore, during colonization, the slight decrease in the TA and organic acid contents is mainly related to infection-induced ethylene production, accelerating the TAC cycle of the fruit [15].

### 3.4. Effects of P. expansum Colonization on the Contents of Fatty Acids in the Different Regions of Apple Fruit

**Figure 6:**
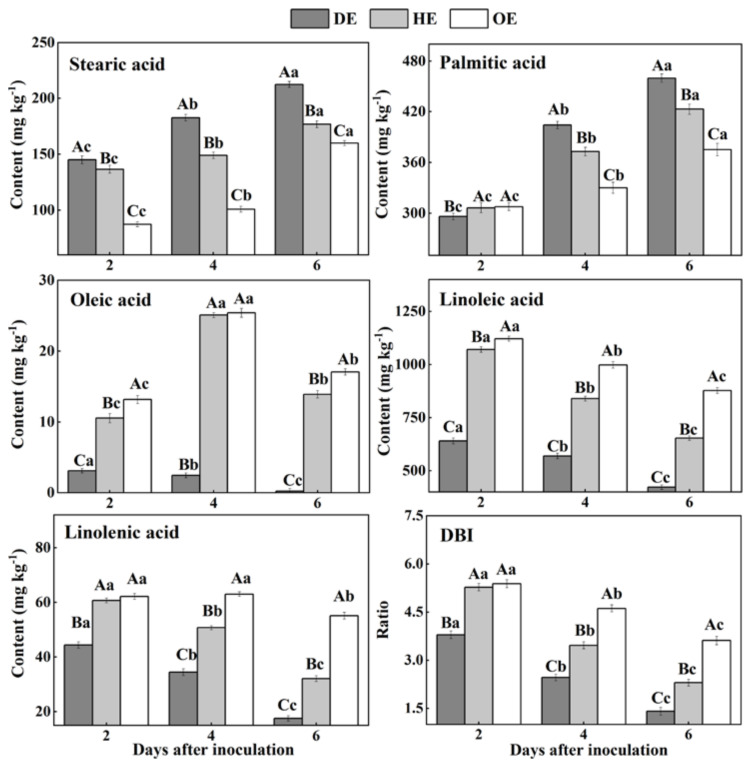
The contents of fatty acids in different regions of *P. expansum*-inoculated apples during incubation. DE: the decayed tissue at the leading edge; HE: the healthy tissue at the leading edge; OE: the healthy tissue 6 mm away from the leading edge. The error bar indicates the standard error (±SE). Capitals indicate a significant difference between different groups at the same time point, and small letters indicate a significant difference in the same group at the different time points (*p* < 0.05).

Fatty acids are an important component of cell membranes [20]. Our results showed that SFAs, stearic acid and palmitic acid accumulated from the distal healthy region to the decayed region (Figure 6). The stearic acid content in the DE region was 22.5% and 81.1% higher than that in the HE and OE regions by the 4th day of inoculation, respectively. Similarly, the palmitic acid content in the DE region was 8.7% and 22.6% higher compared with the HE and OE regions by the 6th day of inoculation, respectively. On the contrary, the content of USFAs, including oleic acid, linoleic acid and linolenic acid, and the DBI values declined from the distal healthy region to the decayed region (Figure 6). The oleic acid content in the DE region was 9.2- and 9.4-fold lower compared with the HE and OE regions by the 4th day of inoculation, respectively. A similar pattern was observed on the 6th day of inoculation, whereupon the linoleic acid content in the DE region was 35.4% and 51.9% lower compared with the HE and OE regions, respectively. Similarly, the linolenic acid content was 45.3% and 68.2% lower in the DE region than the HE and OE regions by the 6th day of inoculation, respectively. Additionally, the DBI value showed a similar pattern, with a 38.8% and 61.3% lower content in the DE region than the HE and OE regions. These results suggest that *P. expansum* may consume the USFAs present in the decayed and healthy regions at the leading edge of the fruit more effectively.

During colonization, the increase in the SFA (stearic acid and palmitic acid) contents and the decrease in the USFA (oleic acid, linoleic acid and linolenic acid) contents can be observed in the decayed tissues. Fungal colonization causes excess reactive oxygen species (ROS) accumulation in the fruit cells, while ROS can trigger phospholipase A2 that catalyzes the membrane lipid, releasing USFAs [51,52]. Moreover, ROS can oxidize USFAs to SFAs, decreasing the degree of unsaturation in the decayed region [53]. The accumulation of stearic acid and palmitic acid in the healthy tissue of the leading edge or that away from the decayed region may be involved in the activation of phospholipase A1 caused by infection [54]. Additionally, the reduction in the contents of linoleic acid, linolenic acid and DBI may relate mainly to the oxidation of USFAs to SFAs by ROS. In the middle stage of colonization, the increase in the oleic acid content in the healthy tissue of the leading edge or that away from the decayed region is mainly due to the activation of lipase activity caused by the infection, which accelerates the hydrolysis of the phospholipid membrane, releasing oleic acid [17]. In addition, compared with the decayed tissue, a higher content of USFAs was observed in the healthy tissue of the leading edge or that away from the decayed region during colonization. Linoleic acid and linolenic acid participated directly or indirectly in the fruit defense via jasmonic-acid-mediated signaling [17,55].

## 4. Conclusions

**Figure 7:**
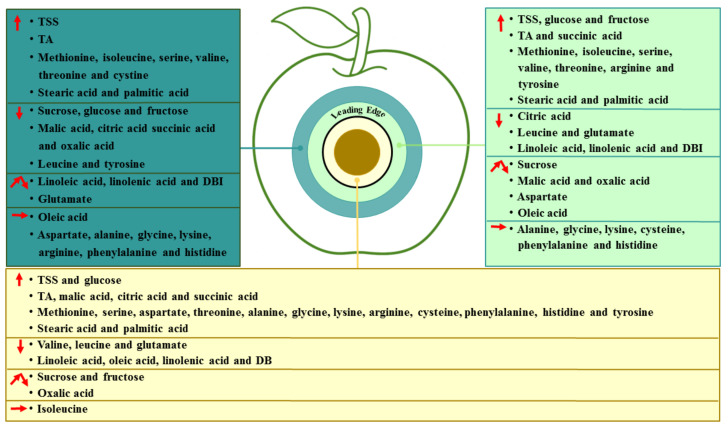
Proposed model of changes in the essential metabolites in different regions of *P. expansum*-colonized apple during incubation. An upward arrow indicates an increase, a downward arrow indicates a decrease, a polyline arrow indicates an increase followed by a decrease and a horizontal arrow indicates no change.

Fungal colonization is a dynamic process, which develops in the colonized sites and spreads to the surrounding healthy tissue of the fruit. Based on the results, we identified changes in the essential metabolites in different regions of *P.* *expansum*-colonized apple during incubation, as shown in Figure 7. *P. expansum* preferred to use sucrose and fructose as carbon sources in the decayed region of the fruit during colonization. The accumulation of fructose and glucose in the healthy tissues of the leading edge may be related to the degradation of starch and sucrose. The breakdown of the vacuole and defense response of the fruit were caused by *P. expansum* colonization, while the decrease in the sugars in the healthy tissues away from the decayed region may be mainly involved in the improved ripening caused by colonization. Moreover, *P.* *expansum* selectively took up amino acids in the fruit as nitrogen sources during colonization. The accumulation of amino acids in the two healthy regions is mainly the result of protein degradation by pathogen-secreted proteases. In addition, the accumulation of organic acids in the decayed tissues is mainly related to the breakdown of the fruit vacuole and accelerated TCA cycle during colonization. The changes in the fatty acid contents in different tissues suggested that the membrane lipid metabolism and ROS production are increased from the distal healthy region to the decayed region, while the defense response of the fruit induced by USFAs may decrease in the same direction. Therefore, *P. expansum* kills cells in advance of its infection of the fruit in order to obtain carbon and nitrogen sources in the distal leading tissue of the infected apple. It is believed that more nutrients are required for the infection and a stronger defense response of the fruit to the fungal infection, causing the transit of nutrients from the distal tissue to the infected sites.

## Data Availability

Data is contained within the article.
